# Comb-locked frequency-swept synthesizer for high precision broadband spectroscopy

**DOI:** 10.1038/s41598-020-59398-1

**Published:** 2020-02-13

**Authors:** Riccardo Gotti, Thomas Puppe, Yuriy Mayzlin, Julian Robinson-Tait, Szymon Wójtewicz, Davide Gatti, Bidoor Alsaif, Marco Lamperti, Paolo Laporta, Felix Rohde, Rafal Wilk, Patrick Leisching, Wilhelm G. Kaenders, Marco Marangoni

**Affiliations:** 1 Dipartimento di Fisica - Politecnico di Milano and IFN-CNR, Via Gaetano Previati 1/C, 23900 Lecco, Italy; 2 TOPTICA Photonics AG, Lochhamer Schlag 19, 82166 Gräfelfing, Germany; 3 Institute of Physics, Faculty of Physics, Astronomy and Informatics, Nicolaus Copernicus University, Grudziadzka 5, 87-100 Torun, Poland; 4 Clean Combustion Research Center, King Abdullah University for Science and Technology, Thuwal, Saudi Arabia

**Keywords:** Near-infrared spectroscopy, Photonic devices

## Abstract

Frequency combs have made optical metrology accessible to hundreds of laboratories worldwide and they have set new benchmarks in multi-species trace gas sensing for environmental, industrial and medical applications. However, current comb spectrometers privilege either frequency precision and sensitivity through interposition of a cw probe laser with limited tuning range, or spectral coverage and measurement time using the comb itself as an ultra-broadband probe. We overcome this restriction by introducing a comb-locked frequency-swept optical synthesizer that allows a continuous-wave laser to be swept in seconds over spectral ranges of several terahertz while remaining phase locked to an underlying frequency comb. This offers a unique degree of versatility, as the synthesizer can be either repeatedly scanned over a single absorption line to achieve ultimate precision and sensitivity, or swept in seconds over an entire rovibrational band to capture multiple species. The spectrometer enables us to determine line center frequencies with an absolute uncertainty of 30 kHz and at the same time to collect absorption spectra over more than 3 THz with state-of-the-art sensitivity of a few 10^−10^ cm^−1^. Beyond precision broadband spectroscopy, the proposed synthesizer is an extremely promising tool to force a breakthrough in terahertz metrology and coherent laser ranging.

## Introduction

For decades, tunable continuous wave (cw) laser sources have been a workhorse for molecular spectroscopy. They may be coupled to optical cavities for highly sensitive absorption measurements, down to 10^−12^ cm^−1^Hz^−0.5^ per spectral point in the near-infrared^1,2^. The advent of optical frequency combs (OFCs) has revitalized the field of cw-laser based spectroscopy, adding repeatability and absolute calibration to the frequency axis and triggering metrological studies on collisional phenomena^3^, molecular quantum models^4,5^ and fundamental constants of physics^6^. Routinely, even in a Doppler broadening regime, comb-assisted spectroscopy (CAS) provides accuracies on the line center frequencies at the kilohertz level, also in combination with enhancement cavities^7,8^.

Alternatively, direct comb spectroscopy (DCS) employs OFCs to directly probe broad absorption spectra and eventually enable detection of multiple gases at high temporal resolution. Several techniques can be adopted to extract the spectroscopic content impressed by the gas on the OFC modes, via dual-comb spectroscopy^9–17^, Fourier Transform spectroscopy (FTS)^18–22^, high-dispersion spectroscopy through Virtually-Imaged-Phased Arrays (VIPAs)^23–26^, or scanning microresonators^27^. In all cases individual comb modes may be addressed, combining the large spectral band with a frequency accuracy ultimately determined by the comb itself.

High sensitivity may also be pursued with DCS, as the evenly spaced spectral structure of frequency combs can be efficiently coupled into enhancement cavities. The best results have been obtained under steady coupling conditions and with optical bandwidths of 3–6 THz, as limited by the dispersion of cavities with finesse in the low 10^4^ range. In most cases the cavity provides an optical filtering of the comb modes that results in a spectral point spacing greater than 1 GHz, eventually preventing a high resolution sampling of absorption lines. An optimized scenario for sensitivity and resolution was achieved years ago by an FTS approach that was capable of covering 3.8 THz with spectral points separated by 380 MHz and a sensitivity on the single point of 3.4 · 10^−9^ cm^−1^Hz^−0.5^ limited by the shot noise on the single comb mode^19^. For high sensitivity and high temporal resolution studies the VIPA approach has offered so far the best performance, with measurement rates of hundreds of hertz and the chance to trigger the acquisition with sub-millisecond resolution^24^. Conversely, as far as the accuracy on line center frequencies is pursued together with high sensitivity, thus for metrological applications, cavity-enhanced-DCS (CE-DCS) has offered so far absolute uncertainties at the MHz level^21,22^, thus almost 3 decades worse than the best CE-CAS results.

In this paper we are proposing a third versatile way to perform spectroscopy with OFCs, which combines the multi-THz spectral coverage of DCS with the 10-kHz level accuracy on the line centers of CAS while keeping the measurement time of seconds over the full band, both with and without an enhancement cavity. Such an approach, hereafter referred to as comb-locked frequency-swept synthesis (CLFSS), is based on the phase locking of a mode-hop-free tunable cw laser to an OFC whose frequency axis is smoothly and seamlessly shifted within the comb spectral range, at speeds up to 1 THz/s and over spectral bands as large as 10 THz. CLFSS ensures precise optical frequency generation and imprints the OFC coherence to the scanning laser, so that both absolute accuracy and narrow linewidth are available for high precision spectroscopy. We assess the performance of CLFSS by studying the 3ν_1_ + ν_3_ band of CO_2_ at a few Pascal pressure over 3 THz, in a cavity with finesse higher than 10^5^ and a noise level on the single spectral point of 6 · 10^−10^ cm^−1^. From fitting an absorption line that was independently measured at NIST with a CAS setup we obtain a transition center frequency in agreement within 30 kHz. A similar agreement is found on the line centers of the ν_1_ + ν_3_ band of C_2_H_2_ investigated in a cavity-free setup.

## Results

### Schematic illustration and technical implementation

In the electrical domain, it is customary to synthesize a single-frequency signal and sweep it over a predetermined spectral range while maintaining frequency and phase coherence with respect to a frequency-calibrated reference input signal. An equivalent synthesizer for the optical domain should adopt an OFC as a reference and a widely tunable cw laser to generate the single-frequency signal; however, a frequency sweep over a broad spectral range in a coherent fashion is not trivial with an OFC because of the ambiguity that arises whenever the cw laser either superimposes a comb mode or lies halfway between two comb modes. Among the solutions proposed so far to solve this ambiguity, methods based on phase locking typically suffer from a small tuning range (tens of GHz) and speed (tens of GHz/s)^28,29^, while much broader (multi-THz^30,31^) and faster (up to 1500 THz/s^32^) methods based on the open-loop calibration of the beat-note signal between an OFC and a scanning cw laser are affected either by coarse frequency calibration^30^ or by the complexity to monitor and process the beat note signal with a phase coherent dual-comb spectrometer^31,32^. Additionally, the open loop regime prevents one to imprint the comb coherence to the scanning laser, to achieve a resolution better than the linewidth of the scanning laser itself and to obtain a fully controlled and reproducible frequency scan. In this work we are overcoming these issues by applying the endless frequency concept of OFC lines proposed by Benkler *et al*.^33^, which enables coherence transfer together with removal of any ambiguity problem by a continuous shifting of the comb lines themselves, while the cw laser follows the shift thanks to a phase lock with a fixed offset to the same comb mode. The proof of principle of the shifter and the characterization of two identical systems were performed a few years ago with the demonstration of mutual coherence of two lasers independently locked to a comb shifting over 28 GHz^34^. Here we propose a substantial leap forward, with an integrated system that offers 100 times larger spectral coverage and 30 times faster tuning speed. These technical advancements allow us to demonstrate for the first time CLFSS-based high precision spectroscopy, both with and without a high-finesse cavity and over entire rovibrational bands of gaseous samples, with state-of-art sensitivity and accuracy.

**Figure 1:**
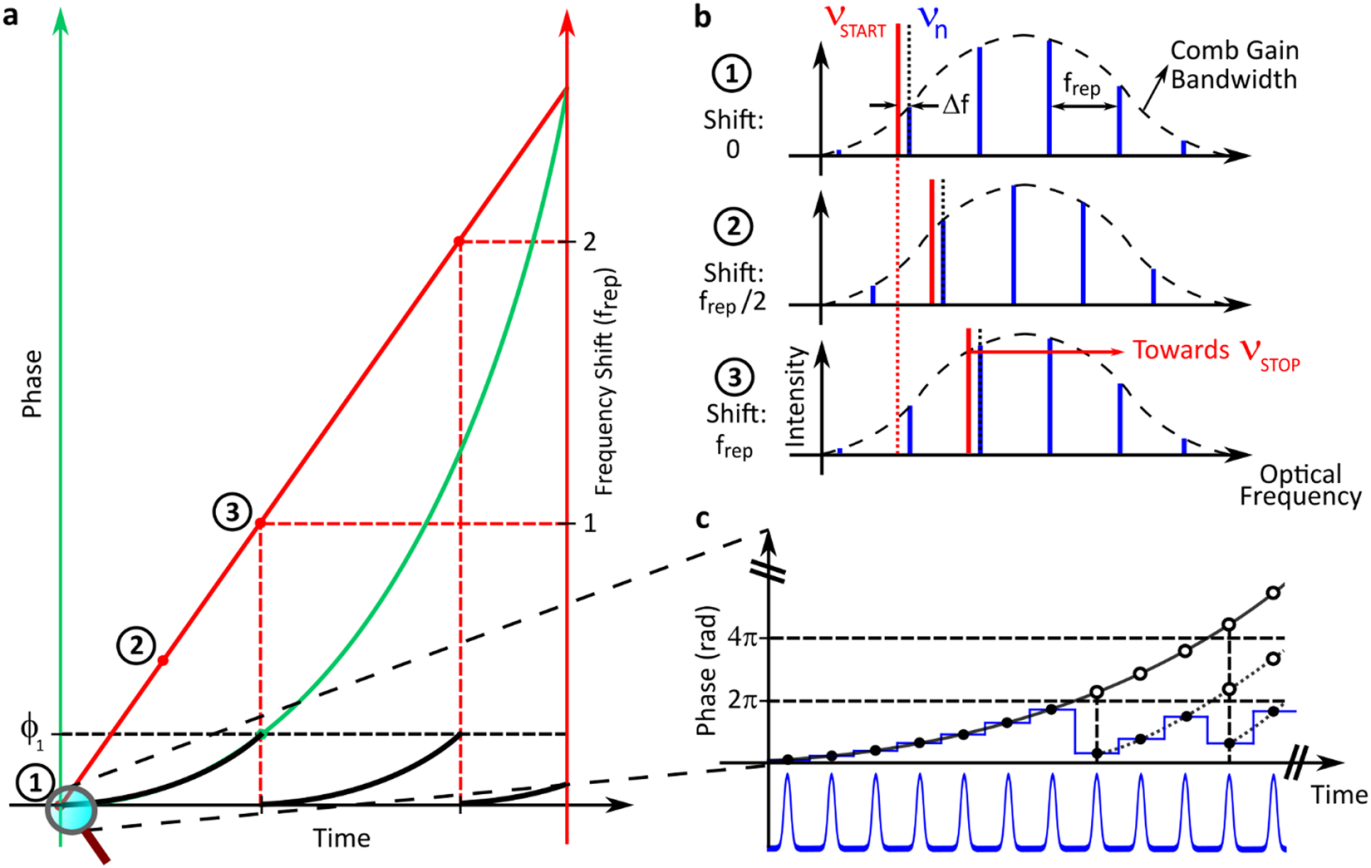
Linear frequency scanning and calibration principle. (**a**) An electro-optic phase modulator applies a quadratic phase shift (green parabola) that determines a continuous linear frequency shift of the comb lines (red line). As the phase advance of 2π per pulse necessary to shift by f_rep_ corresponds mod 2π to a zero phase shift, shifts of multiples of f_rep_ do not contribute to the phase signal, which makes black and green parabolas mod 2π coincident (see Methods for the analytical discussion). (**b**) The uniform spacing (f_rep_) between comb modes makes the comb shifted by f_rep_ (1 → 3 transition) identical to the unshifted comb, while the cw laser has moved by f_rep_. While sweeping from ν_START_ to ν_STOP_ the laser remains phase locked to a comb mode with a fixed offset frequency *Δ*f. (**c**) The phase signal is stepwise applied in between comb pulses, mod 2π to comply with the voltage limitation of the modulator.

The frequency shift of the comb lines is obtained by electro-optic phase modulation. The well-known equality between instantaneous optical frequency and first derivative of the optical phase with respect to time turns a parabolic phase evolution into a linear frequency shift, as it is shown in Fig. 1a and thoroughly described in the Methods. The peculiarity of phase modulation applied to a pulse train that is periodic over time, as it happens with an OFC, is that the phase introduced by the electro-optic modulator (EOM) is sampled at every pulse. This implies that no distortion occurs when phase steps are applied in between two pulses, especially the 2π fly back that overcomes the voltage limitation of the EOM, as it is shown in Fig. 1c. This ensures an endless frequency shifting of the comb and in turn of the cw laser that is locked to one of the comb lines. Phase and frequency deviation of the cw laser has been investigated in detail in Rohde *et al*.^34^. In particular it has been shown to be insensitive to variations of the 2π voltage by a few percent. No aliasing occurs as the radiofrequency beat notes between comb and laser are constant in time and conjugate beat signals never coincide (Fig. 1b).

The sweep is defined by the limiting optical frequencies (ν_START_ and ν_STOP_) and by the scan rate. The tuning speed remains constant in the central part of the sweep and smoothly approaches zero at its edges, thus avoiding phase slips induced by excessive acceleration  of the tunable laser at the turning points. The instantaneous frequency of the laser during the scan is provided by a trigger signal that marks every instant the laser hits the points of a predefined frequency grid. The frequency grid is provided by the same digital electronics that generates the predefined frequency evolution of the scan and corresponding EOM voltage advance. This electronics is clocked by the repetition rate of the comb. Therefore, the time axis and frequency accuracy are derived from the RF reference oscillator, in this case a GPS-disciplined Rubidium clock. The synchronous acquisition of the trigger and of the spectroscopic signal ensures absolute calibration of the frequency axis once the comb order at ν_START_ has been assessed with a wavelength meter.

### Spectrometer layout

**Figure 2:**
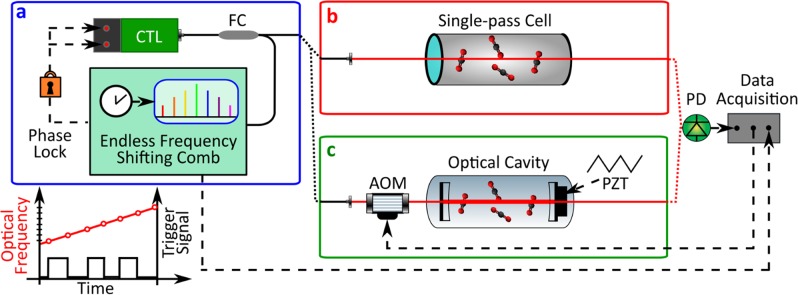
Experimental layout of the Comb-Locked Frequency-Swept Synthesizer for spectroscopy. (**a**) A continuously-tunable laser (CTL) is phase-locked to a self-referenced Er:fiber comb whose frequency is repeatedly shifted over a given range by phase modulation (see Ref. ^34^ for technical details). A trigger signal marks the instants when the laser hits a grid of frequencies that is predefined within the swept range, with a mesh size down to f_rep_/64 in our case. (**b**) Direct absorption spectroscopy setup, composed of cell and detector. (**c**) Cavity-ring-down spectroscopy setup, equipped with an acousto-optic modulator (AOM) to initiate the ring-down decays and a piezoelectric transducer (PZT) to dither the cavity length with a triangular modulation. FC: fiber coupler; PD: photodetector.

The layout of the spectrometer is shown in Fig. 2. An Er:fiber frequency comb (TOPTICA FFS, f_rep_ = 100 MHz) is self-referenced to a GPS-disciplined Rubidium clock through a phase lock of its repetition frequency (f_rep_) in the radiofrequency domain and a frequency lock of its carrier-envelope-offset frequency (f_ceo_). An amplified output of the comb at around 1.55 μm undergoes continuous frequency shifting through a suitable series of phase steps that are applied through a fiber-coupled EOM synchronously with the pulse repetition period (see Methods for details). Whenever the accumulated optical phase shift exceeds 2π, it is folded into the mod 2π voltage range, limiting the necessary voltage applied to the EOM. The electronics that surveys the step-like phase evolution also issues the trigger signal for the frequency calibration, drives the mode-hop-free tunable laser (TOPTICA, Continuously Tunable diode Laser (CTL)) and keeps it phase-locked to the shifting comb by feed-forward control of its grating position and feedback to its piezo and current actuators. The largest spectral range that can be covered by the spectrometer corresponds to the continuous tunability range of the cw laser and comb spectral width, in our case extending over 100 nm from 1525 to 1625 nm.

The cw laser light is either sent along a path to a single-pass cell containing the gas under test (Fig. 2b) or coupled into an optical cavity for high-sensitivity measurements (Fig. 2c). In the first case the transmitted light is collected by a photodetector and sampled in coincidence with the trigger signal for frequency assignment. In the second case the probe laser is mode-matched and then coupled into a cavity with a finesse greater than 10^5^. Every time the scanning laser overlaps a cavity mode, energy is fed into the intra-cavity field giving rise to light transmission. When the transmitted power overcomes a predetermined threshold, an acousto-optic-modulator (AOM) switches off the input laser beam and initiates, at the cavity output, the exponential light decays that are fitted for the retrieval of the intra-cavity absorption, as it is typical in cavity-ring-down spectroscopy^35^ (CRDS, see Methods for details). A slow dithering of the cavity length by means of a piezoelectric transducer varies the position of the cavity modes from scan to scan and provides a simple way to interleave consecutive spectra. During the measurements, the cavity temperature is actively stabilized around 27.5 °C.

### Spectroscopic results

**Figure 3:**
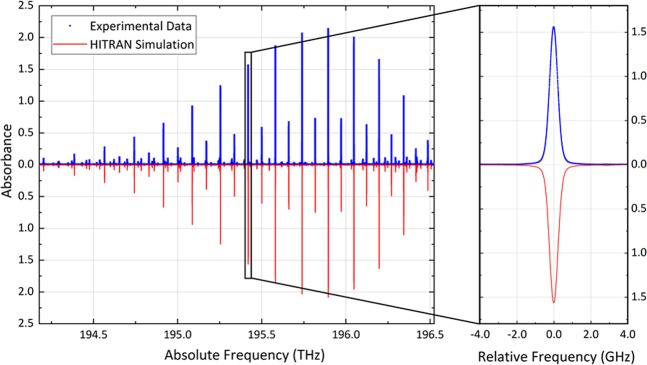
Broadband acetylene absorption spectrum. The measured spectrum (blue dots) extends over 2.3 THz and covers the P branch of the ν_1_ + ν_3_ band of C_2_H_2_ at 1333 Pa. It is the average of 27 consecutive scans at a speed of 341 GHz/s with a spacing of 3.125 MHz between adjacent spectral points, corresponding to 1/32 of the repetition frequency (100 MHz). The overall measurement time is <5 minutes. The frequency scale is absolute and referred to a GPS standard while the vertical scale is in absorbance units, defined as −ln(*I*/*I*_0_) = *αL*, and is consistent with an interaction length of 12.5 cm. The red line is a simulation from HITRAN^36^. The right panel illustrates an 8 GHz-wide cut-out of the spectrum around the P15e line. Note that the R branch was not measured because of the low power spectral density of the comb above 196.5 THz.

We chose molecules such as acetylene and carbon dioxide to benchmark the metrological potential of CLFSS as their line positions in the near-infrared are among the most accurately measured at metrological institutes. Our first experimental tests focused on the P branch of the ν_1_+ν_3_ band of C_2_H_2_, whose lines are sufficiently strong to be observed at a pressure of a few thousands Pascal in a single-pass cell. The absorption spectra were measured by repeating back and forth sweeps over 2.3 THz at variable speeds up to 455 GHz/s. Figure 3 reports an averaged spectrum at a pressure of 1333 Pa and at a speed of 341 GHz/s (blue line, top panel), in excellent agreement with HITRAN^36^ simulations (red line, bottom panel) even by zooming in on a single line (right panel).

**Figure 4:**
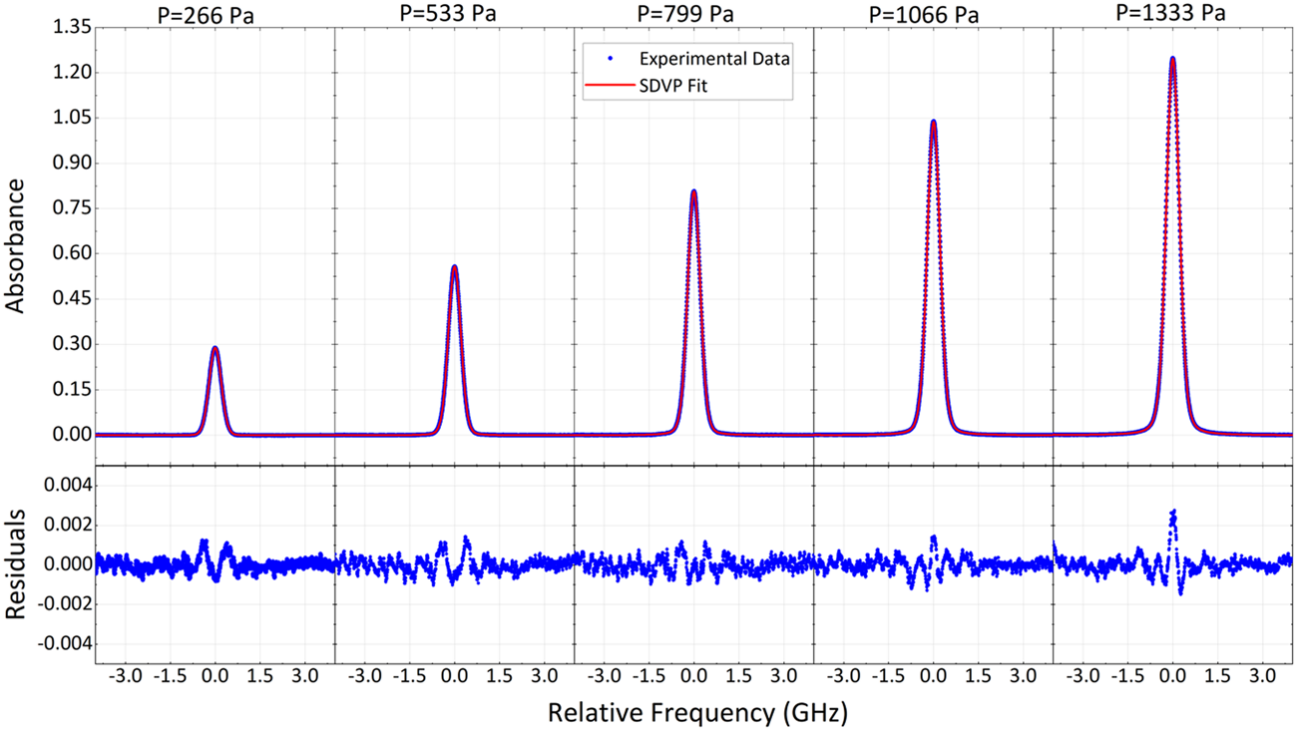
Pressure dependent spectrum of a single acetylene line and multi-spectrum fitting. The spectra refer to the P17e line of the ν_1_ + ν_3_ band of C_2_H_2_ at 5 different pressures, from 267 to 1333 Pa. Each spectrum averages 1110 consecutive spectra acquired with a spectral spacing of 6.25 MHz at a speed of 68 GHz/s over a range of 8 GHz, corresponding to an effective measurement time of 147 s. The total measurement time is 274 s (<5 minutes) due to the dead time (nearly 120 ms) at every turning point of the sweep. A multi-spectrum fitting of the dataset with a speed-dependent Voigt profile^43^ (SDVP) returns a line center value of 195254587021(38) kHz which agrees with that provided by Madej *et al*.^37^ (195254587067.1(2.6) kHz) within 46 kHz. The retrieved pressure shift, −7.40(13) · 10^−3^ cm^−1^/atm, is also consistent with literature data^44^ within 1 standard deviation.

To place a quantitative bound on the accuracy of the spectroscopic determinations, we took advantage of the unique versatility of CLFSS to explore over a narrower spectral range a single line at different gas pressures. Due to the high measurement speed it was possible within a few minutes, i.e. before the onset of drifts related to the pressure leakage of the cell, to average a very large number of spectra and shrink the statistical uncertainty to a few tens of kHz. Figure 4 shows averaged absorption spectra of the P17e line at five different pressures together with the residuals from a multi-spectrum fitting that allowed us to extrapolate the line center frequency without relying on any pressure shift coefficient taken from the literature, thus in a completely independent way. The retrieved position agrees with the highly accurate sub-Doppler determination obtained on the same line at metrological institutes^37^ within 46 kHz, corresponding to 1.2 times our statistical uncertainty (38 kHz).

**Figure 5:**
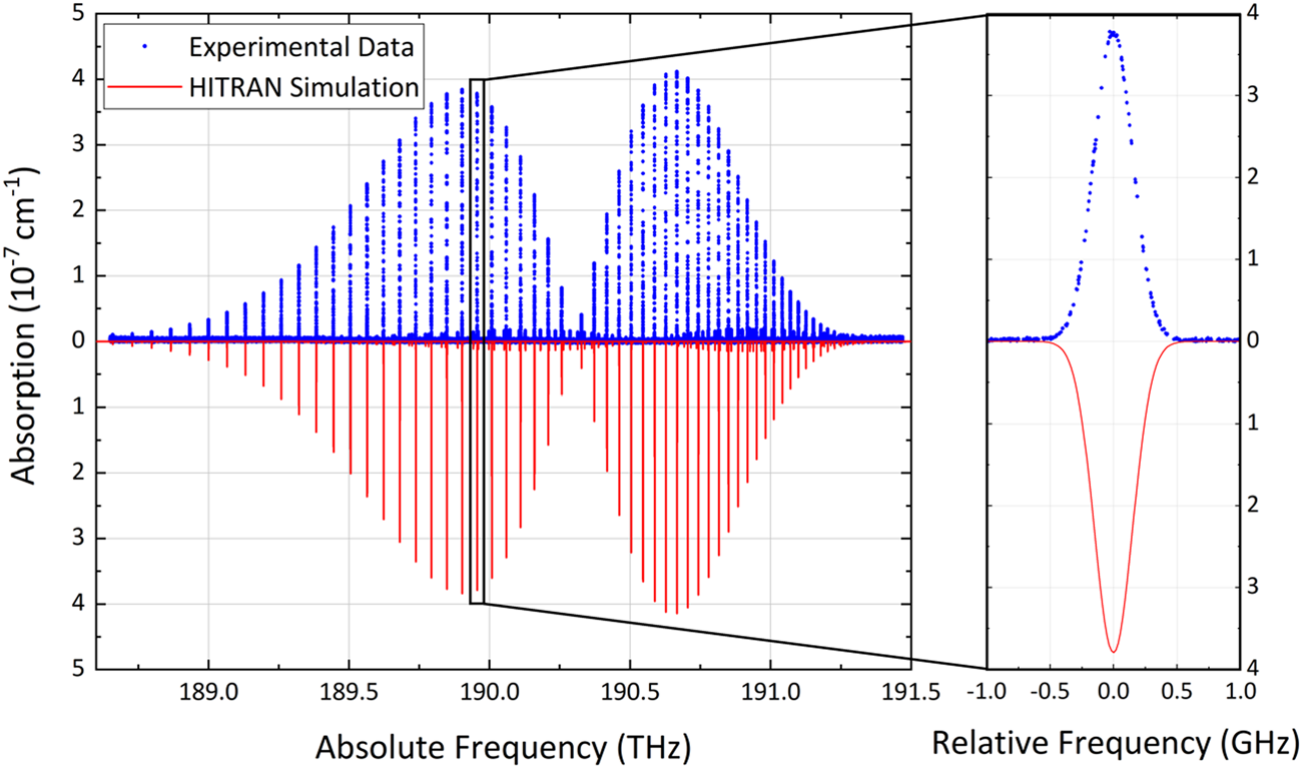
Broadband cavity-ring down absorption spectrum of carbon dioxide. The measured spectrum (blue dots) and the corresponding HITRAN simulation (red line) refers to the 3ν_1_ + ν_3_ band of CO_2_ at a pressure of 1.6 Pa. It has been obtained by interleaving 37 spectra acquired consecutively at a speed of 171 GHz/s over less than 10 minutes. The frequency markers have been set to 1.5625 MHz, corresponding to an effective sampling rate of the ring-down decays of 109 kS/s. The total number of spectral points is 330000 and results from a spectral range of 2.7 THz scanned 37 times with a spacing of 295.5 MHz, as set by the cavity free-spectral-range. The right panel shows a 2 GHz-large cut-out of the spectrum around the P14e line with the corresponding HITRAN simulation (red line).

In order to check the suitability of CLFSS for high sensitivity measurements we focused on the less intense overtone lines of CO_2_ at a pressure of a few Pascal, where absorption profiles are dominated by Doppler broadening and exhibit a nearly Gaussian line shape with full-width-at-half-maximum of 353 MHz only. The gas was housed in the enhancement cavity and its absorption spectrum was directly extracted from the measurement of the ring-down times, thus without any preliminary calibration of the cavity length. The explored range of 2.7 THz is nearly 10 times larger than that achievable in a cavity with similar finesse by CE-DCS, because the cw probe beam is insensitive to cavity dispersion. Figure 5 shows the spectrum of the 3ν_1_ + ν_3_ band repeatedly swept at a speed of 171 GHz/s. The spectral features appear well resolved as the spacing between spectral points is below the free spectral range of the cavity: at every sweep, a slow dithering of the cavity length slightly detunes the frequency axis and produces a straightforward interleaving of spectra.

**Figure 6:**
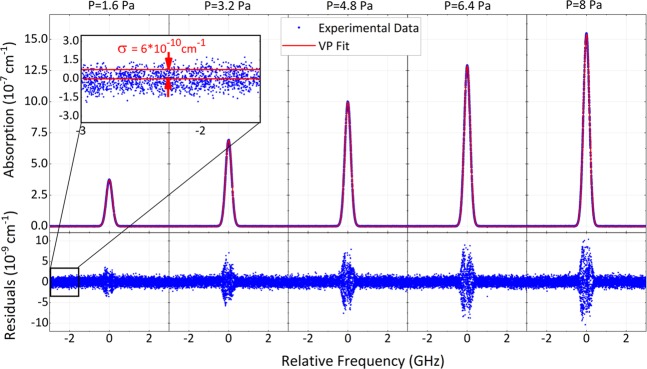
Pressure dependent cavity-ring-down spectrum of a single CO_2_ line. The spectra refer to the P14e line of the 3ν_1_+ν_3_ band of CO_2_ in the 1.6–8 Pa range. The frequency sweeps have been set over 6 GHz and repeated 285 times at 227 GHz/s, corresponding to an effective measurement time of 7.5 s, which further reduces to 1.5 s if one considers that ring-down decays occurring at every 1.3 ms are fitted over 250 μs only (4 times the ring-down-time). The total measurement time, due to dead times at turning points, is 58 s. A multi-spectrum fitting with a Voigt profile (VP) returns residuals with a rms deviation of 6 · 10^−10^ cm^−1^ on the wings of the spectrum, where the signal-to-noise ratio is higher because of the longer ring-down time. The line center retrieved (189955768025(25) kHz) is in excellent agreement with a more accurate measurement performed at NIST^7^ (189955768023(9) kHz).

The absorption profiles well match HITRAN predictions for all lines. The noise level per spectral point (better evidenced by residuals in Fig. 6) is 6 · 10^−10^ cm^−1^. This was limited by our acquisition chain that provided sampling of the ring-down transients at ~110 kS/s, well below the Nyquist limit of 1 MS/s set by a detector bandwidth of 0.5 MHz. As discussed in the Methods and shown in Supplementary Fig. 1S, with a higher sampling rate, in the same cavity and with the same ring-down signal, a noise level of 1.2 · 10^−10^ cm^−1^ is routinely found. This value, as weighted by the square root of the number of spectral points acquired per second (526), returns an equivalent sensitivity of 6 · 10^−12^ cm^−1^ Hz^−0.5^, which compares well with the state of the art of CE-DCS^19^. The major drawback, in our case, is the generally longer acquisition time over a broad band, but this is compensated by the advantage to freely tune, compress or stretch the spectral range, either to locally enhance the sensitivity or to maximize the spectral extension, without any bound set by the interplay between cavity finesse and dispersion.

Figure 6 reports, similarly to Fig. 4, the measurement at different pressures of a single line, namely the P14e, which was characterized in detail both at NIST and at our laboratory with different setups. Each spectrum is composed of 285 consecutive acquisitions. A multi-spectrum fitting with a Voigt profile was found sufficient to obtain featureless residuals within the noise level, which increases around the line peak because of the shorter ring-down time. The extrapolated zero-pressure line center of 189955768025(25) kHz is in agreement with previous highly accurate determinations^7,38^ well within our statistical uncertainty of 25 kHz. This indirectly provides an upper limit also for our systematic errors. As a comparison, this is almost 40 times better than recent results obtained by CE-DCS^21^. It is worth noting that an analysis of line centers separated for back and forth spectra and documented in Supplementary Fig. 2S reveals an offset of ~700 kHz due to delays from the photodetector, the analog-to-digital-converter of the acquisition board, the optical cavity itself (see Methods for details). Those delays remain unchanged in the two directions, however, and they can be easily compensated for by counter-translating the two spectra by half their offset.

## Discussion

We have introduced an approach to precision broadband optical spectroscopy that uniquely bridges the gap between comb-assisted and direct-comb spectroscopy. Its strength resides in the robustness and predictability of phase modulation combined with an optical frequency comb, which is shown to produce continuous and controllable linear frequency shifts over THz ranges. Shown here is the capability to perform spectroscopy with uncertainties at the level of 30 kHz, even in combination with optical cavities for enhanced detection sensitivity. The approach is extremely versatile as the tuning range of the laser is a free parameter, which may be adjusted to cover either an entire rovibrational band or one preselected line repeatedly observed to increase its signal-to-noise ratio, thus matching the ultrabroadband capability of DCS with the accuracy of CAS. For cavity-enhanced measurements, the absence of any lock with respect to the cavity has the twofold advantage of overcoming the trade-off between frequency span and cavity finesse that occurs with CE-DCS due to cavity dispersion and of increasing the spectrometer robustness for field-deployed measurements. In this respect, also the absence of a long mechanical delay line, as compared to FTS^19^, or of a second comb and a second cw laser, as compared to the dual-comb approach^17^, is a significant advancement.

In view of realizing a compact multi-species trace-gas sensor based on a single detector the system could be simplified by removing the comb stabilization, i.e. exploiting the intrinsic stability of the comb-like spectrum of a free-running Erbium oscillator for the frequency calibration of the cavity mode spacing and thus of the measured absorption profile. The tuning range of the external-cavity diode laser here adopted, from 1525 to 1625 nm, is already sufficent to track a variety of gases – such as CO_2_, CH_4_, NH_3_, CO, H_2_S and C_2_H_4_ - and some of their isotopologues at environmental concentrations and with high signal-to-noise ratio. Other species could be detected by a change of the wavelength range of the cw laser and by a concurrent nonlinear extension of the emission range of the comb (through, e.g., second harmonic generation, self-phase modulation or difference frequency generation). This is quite straightforward in the near-infrared, where commercial cw laser solutions are already available in the 0.5–2 μm range, but it would be less trivial in the mid-infrared, mostly because of a cw regime that is not favorable to maximize conversion efficiency and bandwidth of nonlinear optical processes. An accessible solution for the 2.8–5.6 μm range is the difference frequency generation^39^ between a powerful pump laser at 1 μm locked to the comb via supercontinuum generation and a weaker signal laser with tunability in the 1.3–1.7 μm range locked to the same comb via CLFSS. It is worth noting that the chance of using very high-finesse optical cavities in the near-infrared with no restriction on the optical bandwidth strongly mitigates the need for a mid-infrared system: this is attested by the current market of gas analyzers, which is populated to a large extent by cavity-enhanced near-infrared spectrometers that offset the reduced absorption strength of overtone bands by interaction lengths of several tens of kilometers. Moreover, the near-infrared range and the related fiber technology offers a unique chance for cost-effectiveness, portability and robustness.

As s a universal source for a single tunable optical frequency that can be traced back either to an optical or to a radio frequency reference, other applications may be devised for CLFSS. In coherent laser radar systems, for instance, linear frequency modulation of a cw laser is typically exploited to measure distance and velocity of a moving target, with a resolution ultimately limited by modulation linearity and laser linewidth^40^. CLFSS would outperform existing solutions thanks to the intrinsic linearity of the comb frequency scale and to the high degree of temporal coherence that the comb could inherit from a reference laser or cavity. Another front is represented by terahertz metrology, where one could exploit difference frequency mixing in photoconductive emitters^41^ between a CLFSS source and a cw laser locked to the unshifted comb as a broadly tunable, comb-calibrated highly coherent source of terahertz radiation. This would enable high-precision and high-resolution spectroscopy in a spectral region where the calibration of optical frequencies is not straightforward with current technology.

## Methods

### Optical frequency synthesis

The optical synthesizer is based on an external carrier-frequency shifter simultaneously shifting all modes of a comb spectrum. It is combined with a cw laser optically phase locked to one of the comb modes at a fixed frequency offset. The cw spectroscopy laser phase-coherently follows a predefined frequency shift *Δν(t)* within a tuning range that extends over the common spectrum of laser (TOPTICA CTL) and comb (TOPTICA FFS). Since the instantaneous frequency *ν(t)* is shifted from the carrier *ν*_0_ by *ϕ(t)*: $v(t)={v}_{0}+\Delta \nu (t)={v}_{0}+\frac{1}{2\pi }\frac{d\phi }{dt}$, a continuous linear frequency shift *Δν(t)* = *vt* with a tuning rate *v* requires a phase evolution *ϕ(t)* = *πvt*^2^. This is provided by an EOM through phase steps applied in between subsequent pulses as shown in Fig. 1c, thus synchronously with the pulse timing *T*_*rep*_ = 1*/f*_*rep*_. As an example, at *v* = 100 GHz/s, a frequency shift *Δν* = *f*_*rep*_ = 100 MHz is accomplished in a time *T*_1_ = *f*_*rep*_*/v* = 1 *ms* and involves (Fig. 1a) an accumulated phase *ϕ*_*1*_ = *πvT*_*1*_^2^ = 10^5^ π. The phase advance is applied mod 2π to comply with the voltage limitation of the EOM^33,34^. For a further shifting of the comb, e.g. from *f*_*rep*_ to *2f*_*rep*_, the required phase signal *ϕ(t)* is mod 2π identical to that needed to move it from 0 to *f*_*rep*_, apart from the initial phase offset. Seamless shifting is achieved by a phase signal that can be viewed as either a continued green parabola or equivalent recurring black parabolas (see Fig. 1a). This can be understood by observing that a shift by *f*_*rep*_ corresponds to a fully transparent phase step of *Δϕ* = 2π mod 2π = 0 at every pulse (i.e. at every *T*_*rep*_), in other words the phase signal is insensitive to shifts by multiples of *f*_*rep*_ due to the translational symmetry of the comb spectrum. This feature prevents any accumulation of frequency errors upon scanning across multiple *f*_*rep*_. Cumulative phase errors are additionally quenched, as any residual error in the 2π-voltage is reset to zero at each 2π fly back. Since the applied phase progression is synchronized with the repetition rate of the pulse train, the 2π fly backs are applied inbetween pulses and do not contribute to spurious signals in the shifted output. Residual spurious signals originate from imperfections, e.g., of the scaling of the 2π-voltage and thus of the fidelity of the generated voltage signal. By proper optimization they can be suppressed by >30 dB with respect to the carrier. The corresponding phase deviation, shown to be of the order of 100 mrad^34^, is only transferred to the cw laser when the spurs are within the locking bandwidth. The control bandwidth is typically around 1.5 MHz and it is sufficient for phase-slip-free tuning at speeds up to 1 THz/s provided that the signal-to-noise-ratio of the beat-note signal between comb and cw laser is beyond 40 dB in a 100 kHz bandwidth. This condition is fulfilled thanks to a tunable optical bandpass filter that remains centered with respect to the scanning laser wavelength for any combination of scanning parameters and over the whole tuning range of the laser^42^. The linearity of the frequency sweep was experimentally measured at the 2 · 10^−6^ level in the uniform speed region of a 3 THz scan. This slight nonlinearity does not represent a limiting factor for the frequency accuracy of spectroscopic determinations since the EOM phase control engine generates both the phase advance and the corresponding frequency marker, in such a way to accurately track the effective frequency trajectory. This is demonstrated by the lack of artefacts in the residuals of fitted absorption lines even when lines are measured outside the constant-speed range. Relevant factors for the assembling of final spectra are instead the delays in the driving electronics and in the technical realization of the experiment, including the choice of the sampling rate. The delays can be calibrated, however, by comparing backward and forward scans and do not constitute an ultimate limit for accuracy (Supplementary Fig. 2S and related discussion). For large frequency sweeps the total measurement time is barely affected by the time lost at each reversal of the scanning direction (from 100 to 300 ms depending on the speed), whereas for very narrow sweeps the effective measurement time and the dead time may become comparable: in the case of the single-line spectrum of Fig. 4, for example, no appreciable benefit on the measurement time was found for a tuning speed beyond 68 GHz/s, which is why we selected a speed slower than that used for the multi-line spectrum of Fig. 3. From the technical point of view, the advancement here reported as compared to the proof-of-principle experiment of Rohde *et al*.^34^ consists of a faster and larger tuning of the laser, of a tighter laser-to-the-comb phase locking due to the tunable bandpass filter, of a completely redesigned integration of the phase-shifter with the FPGA-based digital laser controller and of the new software control.

### Sensitivity analysis and frequency calibration in CRDS

The optical cavity has a geometrical length of ~50 cm and is equipped with mirrors with 1 m radius of curvature that provide a finesse of >10^5^ between 1460 and 1590 nm. This results in a free spectral range of 295.5 MHz, cavity mode widths of ~3 kHz and ring-down times longer than 60 µs. In the experimental conditions used for the broad spectrum reported in Figs. 5 and 6, namely a tuning speed from 171 to 227 GHz/s and a laser power before the cavity of about 50 mW, the coupled optical power steadily reaches a threshold of about 2 µW: with this signal the repeated acquisition and fitting of ring-down decays from a given cavity mode returns ring-down times with a rms fluctuation of 20 ns if the sampling is performed at 1 MS/s and 16 bits (ideal case, see Supplementary Fig. 1) and of 103 ns with a sampling rate of 110 kS/s at 14 bits adopted for the measurements. This translates into a projected and actual noise level per spectral point of 1.2 · 10^−10^ and 6 · 10^−10^ cm^−1^, respectively. The adopted sampling rate derives from the choice of sampling the ring-down decays synchronously with the trigger signal sent by the control box for the frequency markers, corresponding to 110 kS/s (145 kS/s) with a tuning speed of 171 GHz/s (227 GHz/s) and a mesh size of 1.5625 MHz (f_rep_/64). Albeit suboptimal, this strategy was necessary to prevent excessive data accumulation. A higher tuning speed or a longer cavity would have the advantage of a higher number of spectral points per unit time, but the drawback of a reduced amount of power injected into the cavity and of a proportionally stronger quantization noise. The tuning speed also impacts on the frequency scale, as the frequency assignment at each ring-down event is made by comparing the instant when the laser overcomes the pre-set intensity threshold at the cavity output with the nearby instants at which the trigger signal marks the reaching of the frequency grid points. Any differential delay in the acquisition of those instants results in a systematic shift of the frequency axis between back and forth spectra. As shown in Supplementary Fig. 2S, the shift amounts to ~350 kHz at 227 GHz/s, corresponding to a differential delay of 1.54 μs. This is the cumulative effect of the delays given by the photodetector (~90 ns), by the analog-to-digital converter used to digitize the cavity transmitted power (160 ns) and by the optical cavity itself (~1300 ns), which acts as a low-pass filter for the transmitted photons (note that the build-up time is shorter than the ring-down time because cavity injection is far from producing a steady state regime in our frequency-swept CRDS conditions). Supplementary Fig. 2S also documents that these delays identically occur (within our statistical uncertainty) for the two scanning directions, thereby producing systematic frequency shifts of equal magnitude and opposite sign in back and forth spectra with no alteration of their quality (see, e.g., residuals in Supplementary Fig. 3S). Those shifts can thus be compensated for by counter-translating back and forth spectra by half the distance between their barycenter. As a final technical remark, the acquisition board used for all measurements is a PCI-eXtensions-for-Instrumentation (PXI) board at 100 MS/s that was triggered by the laser control box to acquire the transmitted light synchronously with the predefined frequency grid. The acquisition of the trigger signal was performed through the digital port (10 ns delay) while the measurement of the cavity transmitted power was performed through the analog-to-digital input port mentioned above. A final comment concerns the impact of the comb noise on frequency precision. In our case the frequency comb was stabilized in the RF domain against a low noise RF source (Rohde & Schwarz, Model SMA100A with low noise option) and the comb mode linewidth measured against a kHz-linewidth laser from NKT (Mod. Koheras) amounted to be 65 kHz, which represents an upper limit for the statistical uncertainty of each spectral point: if we consider N spectral points per absorption line, this translates into a line centre uncertainty of about 65 kHz/sqrt(N), thus at the 10 kHz level for N > 40. It is worth noting that with a comb stabilization in the optical domain, thus with comb linewidths well below sub-kHz, the impact on the line centre would result negligible with respect to the contribution given by the noise on the vertical axis of the measurement.

## Supplementary information

Supplementary information.
