# Supplementary material for: Comb-locked frequency-swept synthesizer for high precision broadband spectroscopy
Source: Sci Rep. 2020 Feb 13;10:2523. doi: 10.1038/s41598-020-59398-1 (PMC7018949; doi:10.1038/s41598-020-59398-1)
Supplement: Supplementary file 1 — Supplementary information. [file 41598_2020_59398_MOESM1_ESM.pdf]

SUPPLEMENTARY INFORMATION: Comb-locked frequency-swept synthesizer for high precision broadband spectroscopy

R. Gotti[1]*, T. Puppe[2], Y. Mayzlin[2], J. Robinson-Tait[2], S. Wójtewicz[1,3], D. Gatti[1], B. Alsaif[4], M. Lamperti[1], P. Laporta[1], F. Rohde[2], R. Wilk[2], P. Leisching[2], W.G. Kaenders[2] and M. Marangoni[1]**

[1]*Dipartimento di Fisica - Politecnico di Milano and IFN-CNR, Via Gaetano Previati 1/C, 23900 Lecco, Italy*

[2]*TOPTICA Photonics AG, Lochhamer Schlag 19, 82166 Gräfelfing, Germany*

[3]*Institute of Physics, Faculty of Physics, Astronomy and Informatics, Nicolaus Copernicus University, Grudziadzka 5, 87-100 Torun, Poland*

[4]*Clean Combustion Research Center, King Abdullah University for Science and Technology, Thuwal, Saudi Arabia*

**riccardo.gotti@polimi.it*

***marco.marangoni@polimi.it*

Supplementary Fig. 1

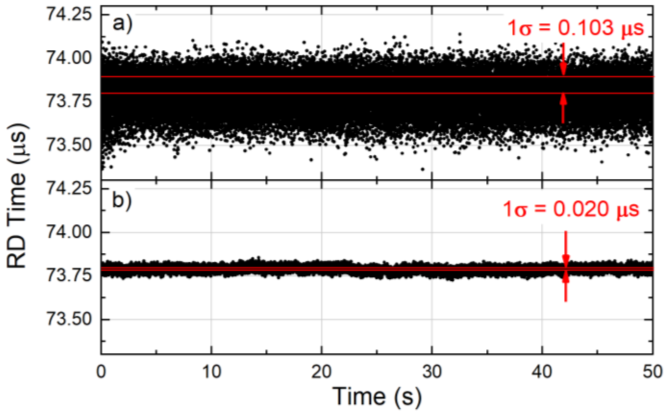

Fig. 1S Sensitivity analysis. a, Sequence of ring-down times measured in empty cavity conditions by CLFSS, with ring-down decays sampled at 145 kS/s and 14 bit depth. The rms fluctuation of ring-down times is 103 ns, corresponding to a limit of detection of $6.3\cdot10^{-10}$ cm^{-1}. The time axis is built from a scanning speed of 227 GHz/s and a cavity FSR of 295.5 MHz, which gives 768 ring-down times per second. A linear baseline has been removed from the dataset because of the dependence of the cavity finesse on the wavelength. **b,** Sequence of ring-down times measured on a single spectral point by cavity length dithering in optimized acquisition conditions, with ring-down decays sampled at 1 MS/s and 16 bit depth. The rms fluctuation of ring-down times decreases by a factor of 5, down to 20 ns, which corresponds to a limit of detection improved to $1.2\cdot10^{-10}$ cm^{-1}. The latter represents a realistic projected value for the sensitivity of CLFSS as the two sequences have been measured with the same initial voltage threshold set for the ring-down decays, thus with the same level of signal and also of detector noise.

Supplementary Fig. 2

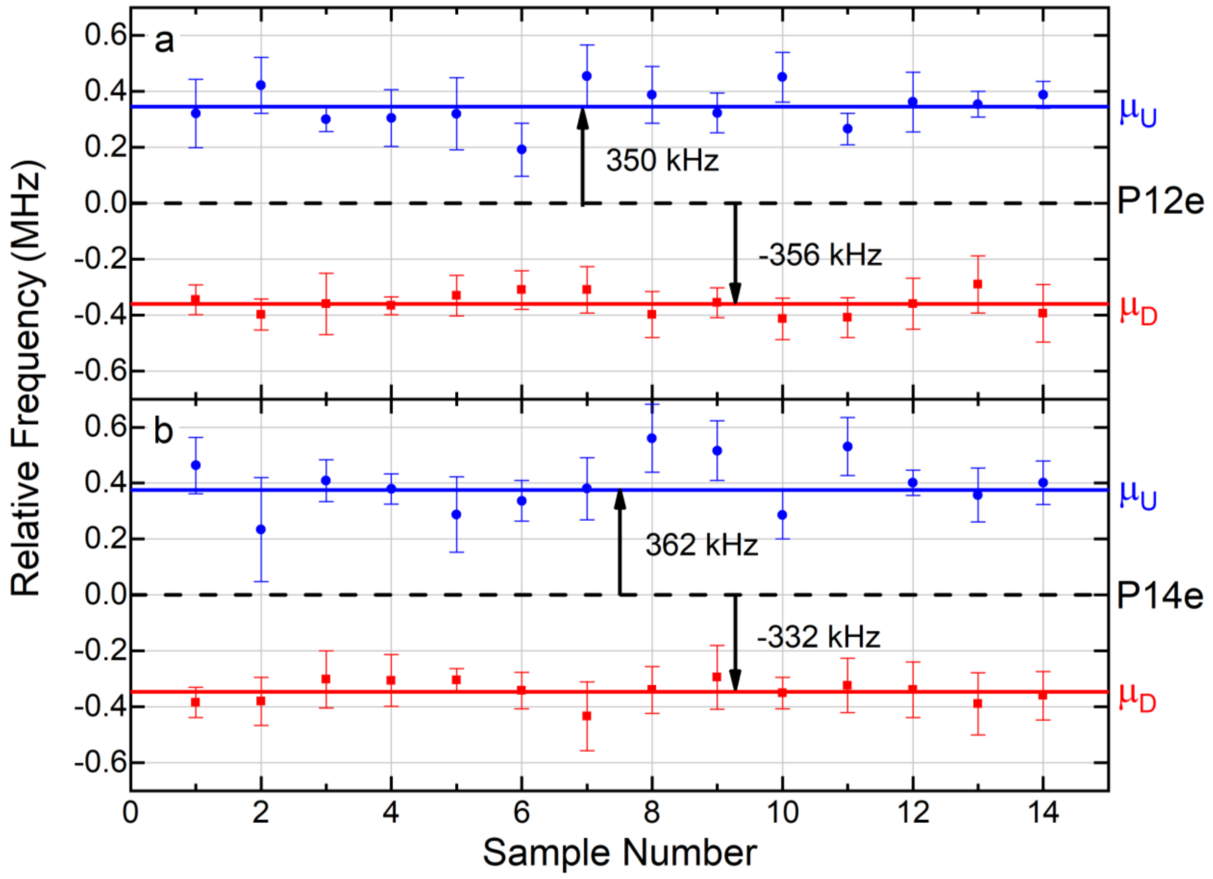

Fig. 2S Center frequencies retrieved with opposite scanning directions for the P12e (panel a) and P14e (panel b) lines of the $3\nu_1+\nu_3$ band of CO_2 (scanning speed: 227 GHz/s and pressure: 8 Pa). Every point is the line center retrieved from the fitting to 10 CRDS spectra acquired with the same speed sign, blue dots referring to increasing (U=upward) scanning frequency and red dots to decreasing (D=downward) frequency. The number of samples and thus of spectra considered is 14 for each line (and for each speed sign). The error bars give the statistical uncertainties of each fit. The offset between averaged line center (μ_U) for U scans and averaged line center (μ_D) for D scans does not depend on the line, as it amounts to 706 and 694 kHz for the P12e and P14e lines, respectively: this proves that the delays of photodetector, ADC converter and optical cavity discussed in the Methods section are the same for the two nearby lines. The comparison of the mean frequencies $(\mu_U + \mu_D)/2$ with the line positions (dashed lines) determined by independent and more accurate measurements (P12: 190008170.181(2)[1] and P14:189955768.022(9)[2]) returns a gap of -3 kHz and 15 kHz for the P12e and P14e line, respectively, thus within our statistical uncertainty. Delays thus equally affect the two scanning directions. Note: the experimental line centers reported in the figure have been extrapolated to zero pressure using the coefficients $-1.586(812)\cdot10^{-2}$ cm^{-1}/atm and $-1.536(776)\cdot10^{-2}$ cm^{-1}/atm retrieved from the multi-spectrum fitting of the P12e and P14e line, respectively.

Supplementary Fig. 3

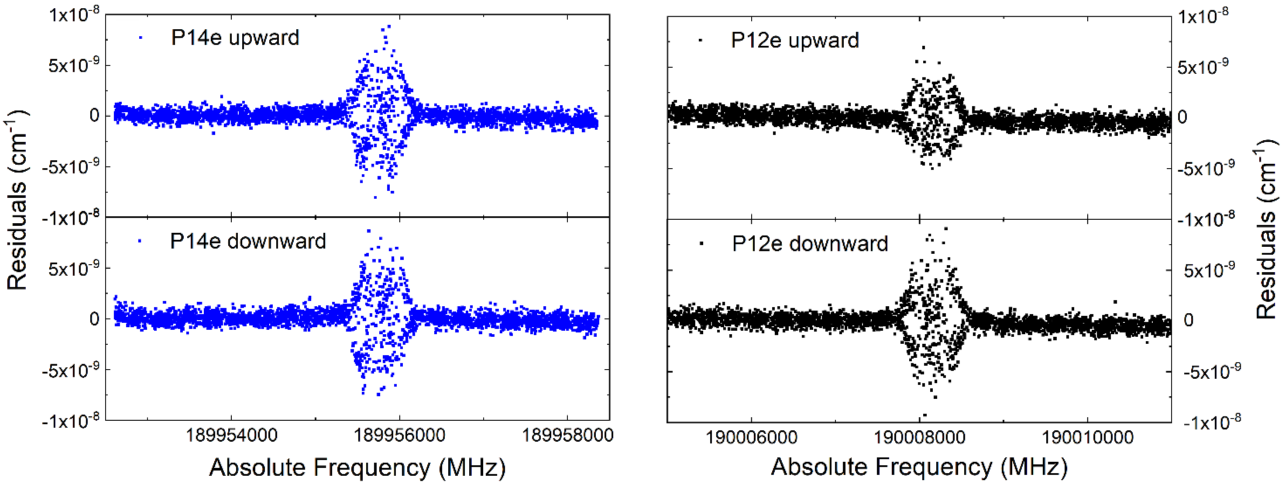

Fig. 3S Residuals obtained from the fit of the P12e and P14e lines for opposite scanning directions (same experimental conditions as for of Fig. 2S). The residuals are calculated from the Voigt fit of 145 interleaved spectra acquired with an increasing scanning frequency (upper panels) and with a decreasing scanning frequency (lower panels). No bias emerges from the noise of the measurement due to different speed signs. The higher noise in the central part around the line peak reflects the lower signal, i.e. the shorter ring-down time, which occurs at higher absorption.

REFERENCES

1. Gotti, R. et al. Conjugating precision and acquisition time in a Doppler broadening regime by interleaved frequency-agile rapid-scanning cavity ring-down spectroscopy. *J. Chem. Phys*. **147**, 134201 (2017).
2. Truong, G.W. et al. Comb-linked, cavity ring-down spectroscopy for measurements of molecular transition frequencies at the kHz-level. *J. Chem. Phys*. **138**, 094201 (2013).
